# Therapeutic Action of the Mitochondria-Targeted Antioxidant SkQ1 on Retinopathy in OXYS Rats Linked with Improvement of VEGF and PEDF Gene Expression

**DOI:** 10.1371/journal.pone.0021682

**Published:** 2011-07-05

**Authors:** Anton M. Markovets, Anzhella Z. Fursova, Natalia G. Kolosova

**Affiliations:** Institute of Cytology and Genetics, Siberian Branch of the Russian Academy of Sciences (SB RAS), Novosibirsk, Russia; University of Oldenburg, Germany

## Abstract

The incidence of age-related macular degeneration (AMD), the main cause of blindness in older patients in the developed countries, is increasing with the ageing population. At present there is no effective treatment for the prevailing geographic atrophy, dry AMD, whereas antiangiogenic therapies successful used in managing the wet form of AMD. Recently we showed that mitochondria-targeted antioxidant plastoquinonyl-decyl-triphenylphosphonium (SkQ1) is able to prevent the development and moreover caused regression of pre-existing signs of the retinopathy in OXYS rats, an animal model of AMD. Here we examine the effects of SkQ1 on expression of key regulators of angiogenesis vascular endothelial growth factor A (VEGF) and its antagonist pigment epithelium-derived factor (PEDF) genes in the retina of OXYS rats as evidenced by real-time PCR and an ELISA test for VEGF using Wistar rats as control. Ophthalmoscopic examinations confirmed that SkQ1 supplementation (from 1.5 to 3 months of age, 250 nmol/kg) prevented development while eye drops SkQ1 (250 nM, from 9 to 12 months) caused some reduction of retinopathy signs in OXYS rats and did not reveal any negative effects on the control Wistar rat's retina. Prevention of premature retinopathy by SkQ1 was connected with an increase of VEGF mRNA and protein in OXYS rat's retina up to the levels corresponding to the Wistar rats, and did not involve changes in PEDF expression. In contrast the treatment with SkQ1 drops caused a decrease of VEGF mRNA and protein levels and an increase in the PEDF mRNA level in the middle-aged OXYS rats, but in Wistar rats the changes of gene expression were the opposite. Conclusions: The beneficial effects of SkQ1 on retinopathy connected with normalization of expression of VEGF and PEDF in the retina of OXYS rats and depended on age of the animals and the stage of retinopathy.

## Introduction

Age-related macular degeneration is the most important cause of impaired vision and blindness in the aging population. But its pathogenesis remains poorly understood. The prevalence of age-related diseases is increasing dramatically as the proportion of the elderly in the population of developed countries continues to rise. Therefore, the development of effective therapeutic and prophylactic agents for AMD is urgently needed. Animal research as well as observational studies suggests that antioxidant supplementation can slow down aging of the eye and possibly provide some protection against AMD [1], [2]. Indeed, oxidative stress is implicated in the aging process and pathogenesis of a wide range of age-related disorders, including AMD. Nonetheless, randomized controlled trials show that antioxidant supplements do not prevent early AMD [3]. There is no evidence that antioxidant supplementation can reduce pre-existing signs of AMD. Moreover, recent human studies reported an increased risk of adverse effects associated with increased intakes of beta-carotene and vitamin E [4]. At present there are tens of thousands of natural and synthetic compounds that possess an antioxidant activity and the number is growing. Correct assessment of antioxidant effects in humans is difficult because of many factors, e.g., the long life span, high cost of randomized controlled trials, ethical issues, and difficult-to-control variables such as quality of life and individual differences in nutrition and age-related health problems. Fortunately, animal models can be used successfully to study biological effects of antioxidants as well as molecular mechanisms and pathways underlying those effects. We showed previously that the senescence-accelerated OXYS rat strain is a suitable model for studying the pathogenesis of AMD and for identifying the relevant therapeutic targets [5]–[9].

The OXYS rat strain was developed at the Institute of Cytology and Genetics, Siberian Branch of the Russian Academy of Sciences, from Wistar stock by selection for susceptibility to cataractogenic effects of galactose as described previously [10], [11].

Progressive mitochondrial dysfunction is considered a possible cause of accelerated senescence in OXYS rats [12], [13] and a possible source of enhanced reactive oxygen species (ROS) production in the tissues of these animals [14]. And, indeed, dietary supplementation with antioxidants can prevent the premature deterioration of mitochondrial function typical for OXYS rats. Recently, it was shown that the mitochondria-targeted antioxidant SkQ1 (plastoquinonyl-decyl-triphenylphosphonium), a conjugate of a lipophilic decyltriphenylphosphonium cation with an antioxidant moiety of a plastoquinone [15], at nanomolar concentrations is capable of preventing some consequences of accelerated senescence in OXYS rats [7]. According to our data, addition of the above-mentioned SkQ1 amounts to food or treatment with SkQ1 eye drops not only completely prevents development of retinopathy but also reduces the severity of pre-existing pathological changes in the retina in OXYS rats [7]. This implies improvement of the animals' vision. It was reported that the effects of SkQ1 include improvement of retinal pigment epithelium functions and reduction of lipofuscin accumulation in the OXYS rat retina [8].

The molecular mechanisms underlying the effects of SkQ1 have yet to be investigated. It is known that in the course of aging, and especially during the development of AMD, an impairment of retinal angiogenesis occurs [16]. It is also known that the key regulators of angiogenesis, vascular endothelial growth factor A (VEGF-A, hereinafter referred to as VEGF) and pigment epithelium-derived factor (PEDF), are involved in the pathogenesis of AMD [17]–[20]. Recently, we reported accelerated reduction of VEGF and PEDF gene expression in the retina of OXYS rats with age in comparison with control (Wistar) rats [9], due to early alterations in RPE cells and choroid vessels in OXYS rats. We supposed that such changes are prerequisite for the development of retinopathy.

The purpose of this study was to assess the effect of SkQ1 on expression of VEGF and PEDF genes in the retina of OXYS rats and to compare it with therapeutic effects of this antioxidant on the signs of retinopathy in these animals. We investigated the prophylactic effect of SkQ1 when added to food between the ages of 1.5 to 3 months, which is the period of manifestation of early signs of retinopathy [9]. In addition, we tested SkQ1 eye drops as a possible therapeutic intervention from age 9 to 12 months, when early alterations in the retina of OXYS rats actively progress to the second stage of retinopathy [9].

## Results

### Ophthalmoscopic examination

**Figure 1:**
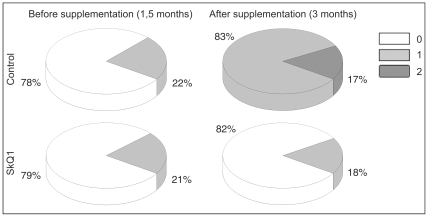
Distribution of OXYS rat eyes by stage of retinopathy. Supplementation with 250 nmol SkQ1/kg per day starting at age 1.5 months prevents the development of retinopathy in OXYS rats. “a.u.” are arbitrary units for estimation of degree of retinopathy (explained in Materials and Methods).

Preliminary examination of rats at the age of 1.5 months showed that the same percentage of eyes in experimental and control groups of OXYS rats had signs of the 1st stage (1 a.u.) of retinopathy (21 and 22%, respectively). SkQ1 supplementation of food (from 1.5 to 3 months of age, 250 nmol/kg) prevented development of retinopathy in OXYS rats (Fig. 1). Therefore at the age of 3 months only 18% of OXYS rats' eyes had signs of the 1st stage of retinopathy, whereas in 82% of eyes the signs of the disease were not detected. In contrast, in untreated OXYS rats retinopathy developed actively, and at the age of 3 months, 83% and 17% of eyes had the signs of the 1st and 2nd stage of retinopathy, respectively.

**Figure 2:**
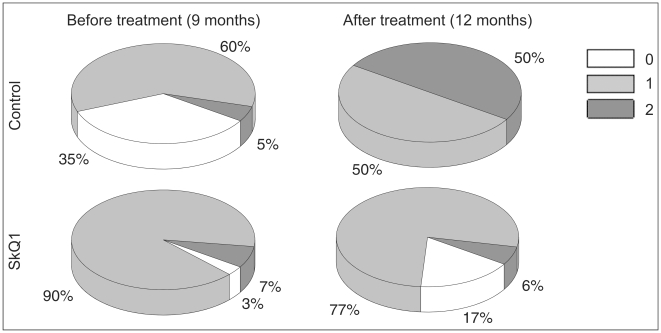
Distribution of OXYS rat eyes by stage of retinopathy. SkQ1 eye drops inhibit the development of retinopathy in OXYS rats, averaged data. “a.u.” are arbitrary units for estimation of degree of retinopathy (explained in Materials and Methods).

When assessing the therapeutic effect of SkQ1 eye drops during the first examination of 9-month-old OXYS rats, all animals had signs of retinopathy in at least one of the eyes. Sixty percent of eyes in the control group (Fig. 2) presented with changes corresponding to the AMD predisciform stage (1 a.u.), 5% corresponding to disciform stage (2 a.u.), and 35% did not have the signs of retinopathy. In the experimental group, 90% of eyes had changes corresponding to the predisciform stage (1 a.u.), 7% corresponding to the disciform stage (2 a.u.), and 3% of rats did not have the signs of retinopathy.

Statistical analysis showed that SkQ1 reduced the severity of pathological changes in the eye-ground of OXYS rats (p<0.000), while in the control animals, retinopathy continued to progress (p<0.0005) (Fig. 2). By the time of the second eye inspection at the age of 12 months, in the control group, the percentage of eyes with changes in the retina corresponding to the 2nd stage of retinopathy (2 a.u.) had increased to 50%, and all eyes had signs of retinopathy. At the time of re-examination of the SkQ1-treated OXYS rats, we did not observe pathological changes in the eye-ground of 17% of eyes, and the percentage of eyes with the 1st stage retinopathy decreased to 77% (Fig. 2).

According to our recent research there is no any evidence of retinopathy in the retina of Wistar rats up to 24 months [9]. The results of this study are consistent with these data. We had not detected any signs of retinopathy in the Wistar rat's retina at the first as well as the repeated inspection of eyes in both experiments (SkQ1 supplementation and SkQ1 eye drops).

### Expression of VEGF and PEDF genes

#### Effects of SkQ1 supplementation of food on gene expression

**Figure 3:**
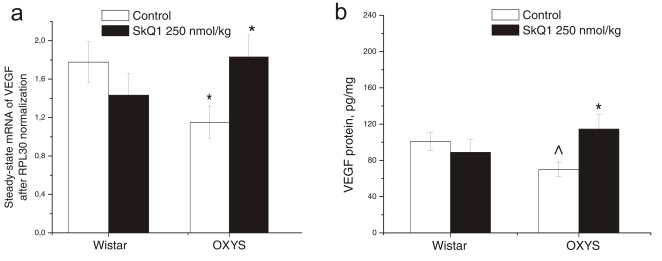
Effects of SkQ1 supplementation (250 nmol per kg of body weight per day) from age 1.5 to 3 months on (a) messenger RNA (mRNA) expression (N = 7) and (b) the protein level (N = 4) of vascular endothelial growth factor (VEGF) in rat retina. Data are presented as mean±SEM (standard error of the mean). * - in comparison with control; ∧ - in comparison with Wistar rats.

Two-way ANOVA showed that VEGF mRNA expression (Fig. 3a) was not affected by genotype (F_1.23_ = 0.27, p = 0.60) and by SkQ1 supplementation (F_1.23_ = 0.58, p = 0.45), but those factors were interacting (F_1.23_ = 5.35, p<0.029). One-way ANOVA showed a decreased mRNA level of the VEGF gene in the retina of control OXYS rats (F_1.12_ = 7.87, p<0.015) compared to the Wistar strain. SkQ1 supplementation did not change VEGF mRNA expression in Wistar rats (p = 0.29), but increased it in OXYS rats (p<0.05) to the normal level of Wistar rats (p = 0.89).

VEGF protein level (Fig. 3b), according to two-way ANOVA, was affected by genotype (F_1.12_ = 7.43, p<0.018) and was not influenced by SkQ1 supplementation (F_1.12_ = 0.69, p = 0.42), although there was a significant interaction between the latter two factors (F_1.12_ = 8.64, p = 0.012). One-way ANOVA revealed a difference between the level of VEGF protein in the retina of untreated Wistar and OXYS rats (Fig. 3b). SkQ1 supplementation increased VEGF protein expression in the retina of OXYS rats (p<0.020) to the level of Wistar rats.

**Figure 4:**
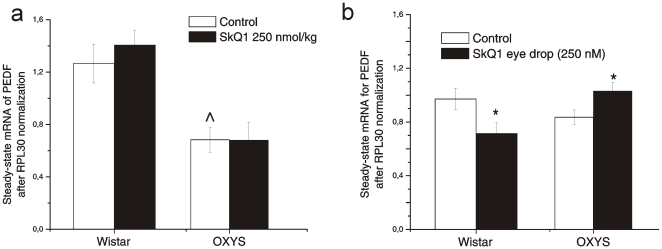
Effects of SkQ1 on retinal level of mRNA of pigment epithelium-derived factor (PEDF), N = 8–10. Data are presented as mean±SEM. **(a)** SkQ1 supplementation, 250 nmol per kg of body weight per day from 1.5 to 3 months of age; **(b)** SkQ1 eye drops (250 nM, one drop per day) from age 9 to 12 months. * - in comparison with control, p<0.05; ∧ - in comparison with Wistar rats.

Expression of PEDF gene (Fig. 4a) was affected by genotype (F_1.27_ = 16.35, p<0.0003) and was not influenced by SkQ1 (F_1.27_ = 0.0003, p = 0.98). The latter two factors did not interact with each other (F_1.27_ = 0.0001, p = 0.99). One-way ANOVA revealed a 47% lower level of PEDF mRNA in the retina of untreated OXYS rats compared to untreated Wistar rats (F_1.13_ = 10.18, p<0.007, Fig. 3a).

#### Influence of SkQ1 eye drops on gene expression

**Figure 5:**
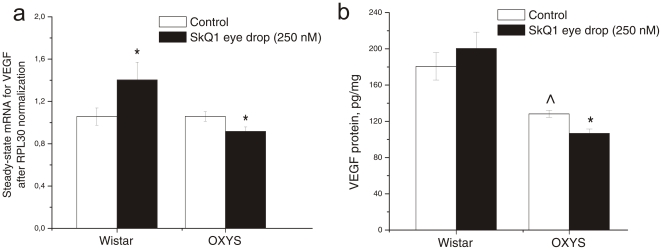
Effects of SkQ1 eye drops (from 9 to 12 months of age) on (a) mRNA expression (N = 8–10) and (b) protein level (N = 4) of vascular endothelial growth factor (VEGF) in rat retina. Data are presented as mean±SEM. * - in comparison with control; ∧ - in comparison with Wistar rats.

Expression of the VEGF gene (Fig. 5a) was affected by genotype (F_1.33_ = 7.48, p<0.001) and was not influenced by SkQ1 (F_1.33_ =  1.32, p = 0.25). A significant interaction between these two factors (F_1.33_ = 7.54, p = 0.001) indicates that the SkQ1 eye drops affected gene expression differently in the retinas of OXYS and Wistar rats. One-way ANOVA analysis revealed no difference between the level of VEGF mRNA in the retinas of untreated Wistar and OXYS rats (Fig. 5a). SkQ1 significantly decreased VEGF mRNA expression in the retina of OXYS rats (F_1.18_ = 5.21, p = 0.034) and there was a tendency for increased expression in the Wistar strain (F_1.15_ = 3.72, p = 0.072). As a result, the level of VEGF mRNA in SkQ1-drop-treated OXYS rats matched the level of untreated Wistar rats (F_1.17_ = 2.50, p = 0.13) and became significantly lower than the VEGF mRNA level of SkQ1-drop-treated Wistar rats (Fig. 5a; F_1.16_ = 9.74, p = 0.006).

A somewhat different situation was observed with the VEGF protein level (Fig. 5b), which, according to two-way ANOVA, depended only on the genotype and was lower in the OXYS retinas (F_1.12_ = 35.16, p = 0.000). One-way ANOVA showed a lower level of VEGF protein in untreated OXYS rats than in untreated Wistar rats. Interstrain comparison showed that SkQ1 eye drops had no effect on the VEGF protein content in Wistar rats but significantly reduced it in the retina of OXYS rats (p<0.017).

Expression of the PEDF gene (Fig. 4b) was affected by genotype (F_1.31_ = 6.93, p<0.013) and was not influenced by SkQ1 eye drops (F_1.31_ = 0.32, p = 0.57). A significant interaction between the latter two factors (F_1.31_ = 14.76, p = 0.000) indicates that the SkQ1 eye drops affected gene expression differently in the retinas of OXYS and Wistar rats. One-way ANOVA revealed no difference between the level of PEDF mRNA in the retinas of untreated Wistar and OXYS rats (Fig. 4b). SkQ1 significantly increased PEDF mRNA expression in the OXYS retinas (F_1.18_ = 5.26, p = 0.033) and decreased it in Wistar rats (F_1.13_ = 11.55, p = 0.004). As a result, the level of PEDF mRNA in SkQ1-drop-treated OXYS rats matched the level of untreated Wistar rats (F_1.16_ = 1.94, p = 0.18) and became significantly higher than the PEDF mRNA level in SkQ1-drop-treated Wistar rats (Fig. 4b; F_1.15_ = 18.42, p = 0.0006).

## Discussion

In our previous study [9] we identified two critical periods for the development of clinical signs of retinopathy in OXYS rats. The manifestation of early disease signs occurs between ages 1.5 and 3 months. Late stage retinopathy develops between 9 and 12 months of age [9]. Additionally, we showed that already at age 20 days the average area of open choroid vessels is decreased while the area of thrombosed vessels is increased in OXYS rats compared to (control) Wistar rats [9]. The developing atrophy of blood vessels leads to hypoxic conditions in the outer retina. Oxidative stress is the main mechanism of tissue damage under hypoxia. In addition, oxidative stress may be both a cause and a consequence of pathological changes in the retina. We found that the mitochondria-targeted antioxidant SkQ1 [15], [21] completely prevents the development of early retinopathy signs in OXYS rats when it is added to food starting from age 1.5 months. Importantly, the antioxidant did not cause any changes in the retina of Wistar rats. The fact that the eyes of the both control and threaded with antioxidant groups of Wistar rats remained healthy indicates that taking of SkQ1 does not have any negative effects. As we showed recently doses of SkQ1 250 nmol/kg for supplementation with food and 250 nM for eye drop are optimal for retinopathy treatment, while 200-fold higher dose (drops contain 25 µM SkQ1) can cause development of new vessels [7].

Same as previously [9], we now show that at age 3 months expression of VEGF and PEDF genes in OXYS rats significantly decreases in comparison with Wistar rats (controls) and it may predispose retina to the development of early retinopathy. It should be pointed out that the differences in mRNA expression of both genes between OXYS and Wistar rats were much greater in the previous [9] than in the present experiment. This difference between the two experiments may be due to differences in the severity of retinopathy. In the previous experiment [9] there were 25% of eyes with 2^nd^ stage and 75% with the 1^st^ stage disease in OXYS rats, whereas now the figures are 17% and 83% respectively. These data support the view that early retinopathy (AMD) is associated with decreased expression of VEGF in the retina. There are many reports in literature that support the existence of a VEGF-mediated physiological mechanism that ensures fine-tuning of choriocapillaris to the necessary nutrient supply to the outer retina [15], [22]. Thus, the premature decrease of VEGF mRNA and protein levels in OXYS rats may cause a disruption of this mechanism and thereby serve as both a cause and a consequence of retinopathy.

We found that, at the molecular level, SkQ1 supplementation prevents a decline of VEGF mRNA expression in OXYS rats and increases protein expression to the normal level. This demonstrates a link between VEGF expression and early retinopathy in OXYS rats. Mitochondrial dysfunction of RPE cells is typical for AMD and accompanies AMD pathogenesis in humans and, one can assume, in rats as well as [23]. Mechanisms of therapeutic action of SkQ1 on retinopathy involve renovation of mitochondrial structure and function in OXYS rats that lead to the reversal of functional insufficiency of retinal pigment epithelium, which produces VEGF and PEDF [7], [21]. These therapeutic mechanisms may also involve the ability of SkQ1 to act as a mitochondria-targeted protonophore [24], which decreases reactive oxygen species (ROS) generation and reduces oxidative damage in ocular tissues. It is likely that SkQ1, acting on mitochondria, blocks mitochondria-mediated apoptosis of RPE cells and photoreceptors and thus prevents the atrophy of this tissue. Beneficial effects of SkQ1 on the endothelium of choriocapillaris cannot be ruled out either.

On the other hand, we did not observe any effects of SkQ1 on PEDF expression at age 3 months—PEDF expression remains low in SkQ1-treated and untreated OXYS rats compared to Wistar rats. It is known that PEDF has anti-angiogenic properties, and therefore we propose that SkQ1 either facilitates the creation of blood vessels or somehow maintains the normal tissue metabolic rate at age 3 months. It should be noted that this is the first study to demonstrate a significant prophylactic effect of an antioxidant compound on retinopathy. Our study also shows the influence of an antioxidant on the expression of genes that are key for retinopathy pathogenesis.

Additionally, we found that SkQ1 can work as a treatment for pre-existing retinopathy in OXYS rats, when it is administered in eye drops starting at age 9 months. In this case, the beneficial effect of the antioxidant also affects the VEGF and PEDF gene expression.

The development of late stage AMD is associated with increased VEGF gene expression, especially in development of neovascularization [25]. We did not observe an increased mRNA level of either VEGF or PEDF at age 12 months—the age of onset of late stage retinopathy—in OXYS rats compared to controls, just like in our previous study [9]. Moreover, the protein level of VEGF is decreased in OXYS rats compared to Wistar. In the present study, we found almost no OXYS rats with detectable neovascularization and this may be explained by the decreased level of VEGF. In addition, we can hypothesize that the VEGF level is locally increased because the amount of RPE cells, which produced VEGF, is decreased according to morphologic analysis [9]. Immunohistochemical analysis would be needed to clarify this question. Bhrutto and coworkers [20] have shown that the cause of the increased VEGF level in late stage AMD is the immune cells that migrate to the retina. It is possible that the development of late stage retinopathy in OXYS rats occurs by the same scenario.

After treatment of OXYS rats with SkQ1 eye drops we also observed changes in gene expression. In OXYS rats, the antioxidant decreased VEGF mRNA and protein levels and increased PEDF mRNA expression. At the same time, VEGF expression (mRNA and protein) increased and PEDF mRNA decreased in Wistar rats. We attribute the interstrain differences in the response to SkQ1 eye drops to the different status of retina in these rat strains. In OXYS rats the development of late stage retinopathy is taking place, and therefore it is biologically necessary to reduce VEGF activity in order to prevent edema and neovascularization. In all experiments determination of different splice isoforms of VEGF-A (for example VEGF_XXX_a or VEGFA –_XXX_b) did not perform and is an aim of further investigation of retinopathy in OXYS rats.

It is likely that SkQ1, which has anti-inflammatory properties [21], suppresses the function of local immune cells (macrophages and others), which are known to release VEGF [17]. In addition, there are reports that SkQ1 prevents macrophage transformation of RPE cells ex vivo [7]. Wistar rats do not exhibit clinical signs of retinopathy, however conform to aging alterations of choroid vessels and RPE occur in the retina of these rats. SkQ1, by increasing VEGF level and decreasing PEDF expression, creates the conditions for survival of choroid vessels in the retina of Wistar rats and prevents the development of retinopathy, as is the case in OXYS rats at age 3 months. This data allow us to suggest that SkQ1 may inhibit development of the retinopathy as one of the possible manifestations of the aging.

One more mechanism that mediates the effects of SkQ1 on VEGF gene expression is the regulation of stability and accumulation of the transcriptional factor hypoxia-inducible factor-1 (HIF-1), which is the main regulator of VEGF expression [17]. Recently, the crucial role of mitochondria-derived ROS for activation HIF-1 was demonstrated and it was shown that the suppression of mitochondrial generation of ROS by SkQ1 effectively blocked HIF-1 accumulation [26]. Additionally, we would not rule out the anti-apoptopic action of this mitochondrial antioxidant, which is mediated by its ability to block the opening of non-specific pores in the mitochondrial membrane.

To date, there is no treatment for early AMD. Currently used anti-VEGF agents typically stop the development of only the wet form of AMD [27]. In the present study, we show that SkQ1 not only prevents the development of early and late stage retinopathy but also causes regression of pre-existing signs of the disease. At the molecular level, we demonstrate normalization of VEGF mRNA and protein levels by means of SkQ1 supplementation. It appears that the effect of SkQ1 on gene expression depends on age of the animals and the stage of retinopathy.

## Methods

### Ethics Statement

All animal procedures were in compliance with the Association for Research in Vision and Ophthalmology statement for the Use of Animals in Ophthalmic and Vision Research as well as the European Communities Council Directive No. 86/609/EES. All manipulations with animals were approved by Scientific Council N 9 of the Institute of Cytology and Genetics Siberian Branch of the Russian Academy of Sciences, according to “The Guidelines for Manipulations with Experimental Animals” (the decree of the Presidium of the Russian Academy of Sciences of April 02, 1980, no. 12000-496).

### Animals

Male senescence-accelerated OXYS and age-matched male Wistar rats (as controls) were obtained from the Breeding Experimental Animal Laboratory of the Institute of Cytology and Genetics, Siberian Branch of the Russian Academy of Sciences (Novosibirsk, Russia). At the age of 4 weeks, the pups were weaned, housed in groups of five animals per cage (57×36×20 cm), and kept under standard laboratory conditions (22±2°C, 60% relative humidity, and natural light), provided with a standard rodent feed, PK-120-1, Ltd. (Laboratorsnab, Russia), and given water ad libitum.

### Studies of SkQ1 effects

SkQ1 was synthesized at the Institute of Mitoengineering of Moscow State University (Moscow, Russia). Rats were distributed among the control and experimental groups by simple random sampling. To study the effects of SkQ1 on the development of retinopathy in OXYS rats we conducted two experiments. In the first experiment, 250 nmol SkQ1 per kg of body weight per day were added to the feed of OXYS and Wistar rats between ages 1.5 and 3 months; the control groups of rats did not receive SkQ1. In the second experiment, 0.9% NaCl solution (control group) or 250 nM SkQ1 in 0.9% NaCl (experimental group) was instilled to the eyes of OXYS and Wistar rats in the amount of one drop day in the form of eye drops from 9 to 12 months of age. In total, 60 animals were used in each experiment, 15 animals in each group (OXYS control, OXYS experiment, Wistar control, and Wistar experiment).

### Ophthalmoscopic examination

**Figure 6:**
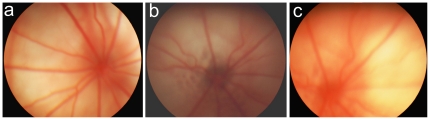
Fundus photograph of rat retina (a) Normal fundus from 12-months-old Wistar rat. Ratio of blood vessels is normal. The retina between vascular arcades is not damaged (changed). (**b**) Geographic atrophy of retinal pigment epithelium of retina from 3-months-old OXYS rats. Obliteration of choroidal vessels, obliteration and sclerosis of retina vessels. Redistribution of pigmentation. (**c**) Fundus photograph from 12-months-old OXYS rat. Confluent soft drusen-like deposits, retinal pigment epithelium detachment, latent choroidal neovascularization on optic disc.

All animals were examined by an ophthalmologist twice, before and after SkQ1 supplementation or eye drop treatment, at the age of 1.5 and 3 months or at the age of 9 and 12 months, respectively. All rats underwent fundoscopy with a “Heine BETA 200 TL” Direct Ophthalmoscope (Heine, Germany) after dilatation with 1% tropicamide. Assessment of stages of retinopathy was carried out according to the Age-Related Eye Disease Study (AREDS) grade protocol (http://eyephoto.ophth.wisc.edu). The degree of retinopathy was estimated as follows: 0 arbitrary unit (a.u.) corresponds to health retina (Fig. 6a); 1 a.u. corresponds to appearance of drusen and other pathological changes in the retinal pigmented epithelium (RPE) and partial atrophy of the choroid capillary layer (Fig. 6b); 2 a.u. means exudative detachment of RPE and of retinal neuroepithelium, with further choroid capillary layer atrophy (Fig. 6c. Five days after the last eye examination, the rats were decapitated and the studies of the effects of the mitochondria-targeted antioxidant SkQ1 on VEGF and PEDF gene expression were performed. Kowa Genesis-D fundus camera (Japan) was used as a hand-held digital fundus camera to take digital fundus photographs of retina.

### Studies of VEGF and PEDF gene expression

Steady-state mRNA expression of VEGF and PEDF was studied in the retinas of the control and SkQ1-treated OXYS and Wistar rats using real-time PCR. There were 7–10 animals in each group. Rats were decapitated and eyes removed. The retina for gene expression analysis was separated from the other tissues, placed in microcentrifuge tubes for RNA isolation and frozen in liquid nitrogen. All specimens were stored at –70°C prior to the analysis.

Total cell RNA was isolated from rat retina using TRI-Reagent (Ambion). The amount of isolated RNA was assessed by means of electrophoresis of 1 µl of each RNA sample in 1.5% agarose gel with ethidium bromide staining. RNA concentration in each sample was determined using spectrophotometry at 260 nm and also by absorbance ratios 260/280 nm and 260/320 nm. RNA was stored at –70°C. Contaminating genomic DNA was removed by treatment with DNase I (Promega, USA) according to the vendor's manual and then by repeated RNA extraction with the phenol-chloroform mixture and pure chloroform, followed by precipitation with propanol. Reverse transcription was performed using M-MLV Reverse Transcriptase (Promega, USA). For subsequent PCR, we used 0.25–1.0 µl of the resulting cDNA solution.

Aliquots (4 µl) from all cDNA samples were mixed and the “average” solution was used for preparation of calibration curves, which were used for determination of a relative cDNA level for genes of interest and a reference gene in experimental samples.

Age-related changes of vegfa and pedf gene expression were studied using iCycler iQ4 real-time PCR detection system (Bio-Rad Laboratories, USA) and SYBR Green I dye (Molecular Probes, USA). The housekeeping gene Rpl30 (encoding large ribosomal subunit protein 30) was used as a reference gene. The following primers were used: 5′_ATGGTGGCTGCAAAGAAGAC_3′ and 5′_CAAAGCTGGACAGTTGTTGG_3′ for Rpl30; 5′_CTGGCTTTACTGCTGTACCTCCACC_3′ and 5′_GGCACACAGGACGGCTTGAA_3′ for vegfa; and 5′_GATTGCCCAGCTGCCTTTGACA_3′ and 5′_GGGACAGTCAGCACAGCTTGGATAG_3′ for pedf.

The reaction mixture (final volume 25 µl) contained the standard PCR buffer (67 mM Tris-HCl pH 8.9, 16 mM (NH_4_)_2_SO_4_, 0.01% Tween 20, and 10 mM β-mercaptoethanol), MgCl_2_ (3 mM for vegf, 1.5 mM for RPL30, and 3 mM for pedf), 0.2 mM dNTPs, SYBR Green I (1∶20,000 dilution), 150 nM of each primer, and 0.8 U of Taq polymerase (Institute of Cytology and Genetics, Russia). Reaction was carried out under the following conditions: heating at 95°C for 3 min (initial denaturation), and then 40 cycles: denaturation at 95°C for 20 sec, annealing at 60°C for 20sec, elongation at 72°C for 30 sec. Data collection was based on fluorescence for Rpl30 at 84°C for 30 sec and for the vegf and pedf genes at 87°C for 10 sec. After the completion of PCR, the melting curves for specificity control were recorded. In each experiment, samples of cDNA under study were mixed with primers for a gene of interest (four repeats per cDNA sample) in one microtube plate; similar samples were mixed with primers for the reference gene (also four repeats). “Standard” cDNA was diluted from 1∶2 to 1∶64 with the same primers (2–3 repeats). For each cDNA sample, PCR was repeated at least twice. To confirm amplicon size and reaction specificity, PAGE electrophoresis was performed with DNA molecular weight markers.

The initial quantitation of cDNA concentration in the samples was carried out using standard calibration curves (versus “standard” cDNA) and the gene expression value was obtained for each gene of interest; this value was then normalized according to the amount of the reference gene cDNA [28].

### Enzyme-Linked Immunosorbent Assay

Protein was isolated from rat retina using TRI-Reagent (Ambion). Enzyme-linked immunosorbent assay (ELISA) for VEGF (RayBiotech, USA) was performed according to the manufacturer's instructions, except that equal protein concentrations were loaded into each well. Quantitation was carried out according to the optical density measurement obtained using a microtiter plate reader and recalculated as pg of VEGF protein per mg of retinal tissue.

### Statistical analysis

The data were analyzed using repeated measures ANOVA with the statistical package Statistica 6.0. Two-way ANOVA was used to evaluate effects of treatment. The independent variables were genotype (Wistar, OXYS) and treatment (controls, SkQ1). A Newman–Keuls post-hoc test was applied to significant main effects and interactions in order to estimate the differences between particular sets of means. One-way ANOVA was used for individual group comparisons. To assess the therapeutic effectiveness we performed dependent pairwise comparison of the eye states before and after treatment (T-test for dependent samples). Data are represented as mean ± S.E.M. Results were considered statistically significant if p value was less than 0.05.
